# Overexpression of Cassava *MeSTP7* Promotes *Arabidopsis* Seedling Development

**DOI:** 10.3390/plants13213102

**Published:** 2024-11-04

**Authors:** Sha Geng, Xiaotong Wang, Wei Yan, Qian Liu, Na Wang, Jianyu Zhang, Jianchun Guo, Jiao Liu, Lijuan Luo

**Affiliations:** 1 Institute of Tropical and Subtropical Cash Crops, Yunnan Academy of Agricultural Sciences, Baoshan 678000, China; rjsgengsha@163.com (S.G.); rjsyanwei@163.com (W.Y.); wtqs123@163.com (Q.L.); wang931341951@163.com (N.W.); 2 National Key Laboratory for Tropical Crop Breeding, Sanya Research Institute, Institute of Tropical Bioscience and Biotechnology, Chinese Academy of Tropical Agricultural Sciences, Haikou 571101, China; wangxiaotong@hainanu.edu.cn (X.W.); guojianchun@itbb.org.cn (J.G.); 3 Key Laboratory of Sustainable Utilization of Tropical Biological Resources of Hainan Province, School of Tropical Agriculture and Forestry, Hainan University, Haikou 570228, China; jianyuzhang@hainanu.edu.cn; 4 School of Breeding and Multiplication (Sanya Institute of Breeding and Multiplication), Hainan University, Sanya 572025, China

**Keywords:** cassava, sugar transporter protein, transgenic *Arabidopsis*, hormone treatment, *MeSTP7*

## Abstract

The sugar transporter (STP) gene family is a key regulator of plant development, which is crucial for the efficient transport and utilization of sugars during plant growth and development. In this study, we identified the *MeSTP7* gene, which is highly expressed in cassava fibrous roots, early storage roots, and under hormonal treatment, including IAA, MeJA, ABA, and GA_3_, and abiotic stressors, such as mannitol and NaCl. A strong response was observed with exoqenous IAA. Transfecting *MeSTP7* into *Arabidopsis* promoted early seedling growth, particularly in lateral root development. The content of endogenous hormones (IAA and MeJA) as well as soluble sugars (sucrose, fructose, and glucose) was elevated in transgenic *Arabidopsis*. Hormone treatments with IAA, MeJA, GA_3_, and ABA on transgenic *Arabidopsis* revealed that transgenic *Arabidopsis* responded positively to added 20 μM IAA. They also exhibited co-induced regulation of lateral root formation by GA_3_, MeJA, and ABA. qRT-PCR analysis showed that overexpression of *MeSTP7* upregulated the expression of *IAA14*, *ARF7*, and *ARF19* in *Arabidopsis*. Under IAA treatment, the expression of these genes was similarly upregulated but downregulated under MeJA treatment. These results suggest that *MeSTP7* may promote *Arabidopsis* seedling development by increasing the content of sucrose, glucose, and fructose in roots, which in turn influences IAA-based hormonal signaling.

## 1. Introduction

Cassava is rich in carbohydrates and can be cultivated in poor soil conditions, making it a valuable source of food and income for over 800 million people in rural areas [1]. It is used to produce a variety of products, such as starch, bioethanol, and other bio-based products, including feedstuffs, pharmaceuticals, cosmetics, and biopolymers [2]. However, its growth and development can be limited by various factors, including soil fertility and planting material quality [3]. Currently, there is a significant deficiency in the exploration of genes that promote the growth and development of cassava, a shortfall that has implications for both agricultural productivity and food security in regions where this crop is a dietary staple. Therefore, it is crucial to explore the genes that promote the growth and development of cassava to unlock their potential and contribute to global food security and sustainable agricultural development.

In most plants, sugar transporters mediate the long-distance transport and allocation of photosynthates from the source-to-sink organs [4,5]. Three key types of sugar transporters exist: sucrose transporters (SUTs), sugars will eventually be exported transporters (SWEETs), and monosaccharide transporters (MSTs) [6]. MSTs are the largest family of sugar transporters and are involved in the transport of a variety of monosaccharide substrates [6]. Generally, the MSTs family, based on sequence characteristics and substrate specificities, can be classified into seven subfamilies: sugar transport protein (STP/HT), polyol transporter (PLT), early response to dehydration (EDRL), plastidic glucose transporter (pGlucT), inositol or cyclic polyol transporter (INT), tonoplastic monosaccharide transporter (TMT), and vacuolar glucose transporter (VGT) [7].

Monosaccharide transport proteins, which belong to the major facilitator superfamily (MFS), are membrane proteins that typically possess 12 transmembrane domains. They are categorized as membrane-associated proteins with an amino-terminal that spans the membrane six times on one side and a carboxy-terminal that spans the membrane six times on the other side [8]. Despite having highly homologous amino and carboxyl-terminal sequences across their transmembrane domains, different monosaccharide transport proteins can exhibit significant functional differences [8]. MSTs are widely present in all plant tissues and cells and serve multiple functions. They can transport, absorb, utilize, and accumulate monosaccharides, affecting plant growth and development by participating in the extracellular offloading of phloem hexose, the carbohydrate supply during pollen development, and by providing energy and developmental signals for plants [9,10,11,12]. It also enhances plant defense and regulates resistance to abiotic stressors, such as drought and cold [13,14,15]. In *Arabidopsis*, 14 MSTs capable of transporting sugars have been identified as *AtSTP1* to *AtSTP14* [16,17]. For instance, *AtSTP2* provides glucose to pollen during pollen development [18], while *AtSTP6* plays a role in the sugar supply during pollen germination and pollen tube growth [19]. *AtSTP1* participates in the regulation of branching in *Arabidopsis* by controlling the expression of genes related to hormone synthesis and signal transduction, thus influencing the sugar signaling pathway [20,21]. In rice, knocking out the sugar transporter protein OsSTP15 increases the sugar content in the lower part of the stem, which in turn increases the number of tillers and enhances grain yield [22]. In rice, *OsMST8* is involved in the response to cold stress by regulating changes in sugar content [23]. The *OsMST6* gene is induced by high sugar and salt stress [24]. The heterologous expression of *MdHT2.2* in apples regulates apoplastic hexose levels in tomato fruit, controlling CWINV activity, altering carbohydrate distribution, and resulting in increased fruit size [25]. Furthermore, expressing *MdSUT2* in tomatoes leads to increased soluble sugar levels in transgenic lines, enhancing the plants’ tolerance to adverse environmental factors such as salinity and drought [26].

Since plants are anchored in the soil and exhibit a sessile growth form, their access to water and essential nutrients relies heavily on the underground portions of the root system. Therefore, the growth and development of roots directly influence plant growth and subsequently affect the plant’s ability to respond to environmental stressors. The root system encompasses the entirety of a plant’s roots. The primary root develops from the seed embryo, and under the combined influence of internal and external factors, the primary root grows multiple lateral branches at specific angles, known as secondary or lateral roots [27]. Many dicotyledonous plants, such as *Arabidopsis thaliana*, *Brassica napus*, *Solanum lycopersicum*, and *Daucus carota*, possess distinct primary roots, with lateral roots developing from the primary root under the influence of endogenous hormones and environmental factors. Although various plant hormones regulate lateral root development, auxin is the most critical growth regulator [28]. In *Arabidopsis*, INDOLE-3-ACETIC ACID INDUCIBLE14 (IAA14) serves as the key transcriptional regulator of auxin signaling during the development of lateral roots [29,30,31]. Gain-of-function mutations in the SLR1/IAA14 gene in *Arabidopsis* can hinder the formation of lateral roots [29]. The SLR1/IAA14 gene encodes a member of the Aux/IAA protein family, which acts as a transcriptional repressor in the auxin signaling pathway. The stabilized mutant form of *IAA14* (mIAA14) in the gain-of-function slr-1 mutant is believed to inactivate the functions of *ARF7* and *ARF19*, which are transcriptional activators in auxin signaling, thereby blocking lateral root initiation [30,32]. Recent studies have shown that jasmonic acid can inhibit the formation of lateral roots induced by auxin, and this inhibitory effect is independent of the jasmonic acid receptor CORONATINE INSENSITIVE 1 (COI1) [33,34]. This suggests that jasmonic acid may regulate the formation of lateral roots by influencing the auxin signaling pathway.

Currently, there are few reports on the involvement of STPs in plant root development. Most studies focus on the role of STPs in fruit and floral organ development, as well as the establishment of stress resistance mechanisms. In this study, we found that overexpressing the cassava *MeSTP7* gene in *Arabidopsis* enhances early seedling growth, especially lateral root development. When treated with the hormones indoleacetic acid (IAA), methyl jasmonate (MeJA), abscisic acid (ABA), and gibberellin A3 (GA_3_), the transgenic *Arabidopsis* exhibited increased sensitivity to IAA and MeJA. IAA promoted lateral root formation in the transgenic lines, while MeJA inhibited their development. These results provide new insights into the role of the *MeSTP7* gene in regulating plant growth and development.

## 2. Results

### 2.1. Expression Patterns of MeSTP7 in Cassava Tissues

**Figure 1:**
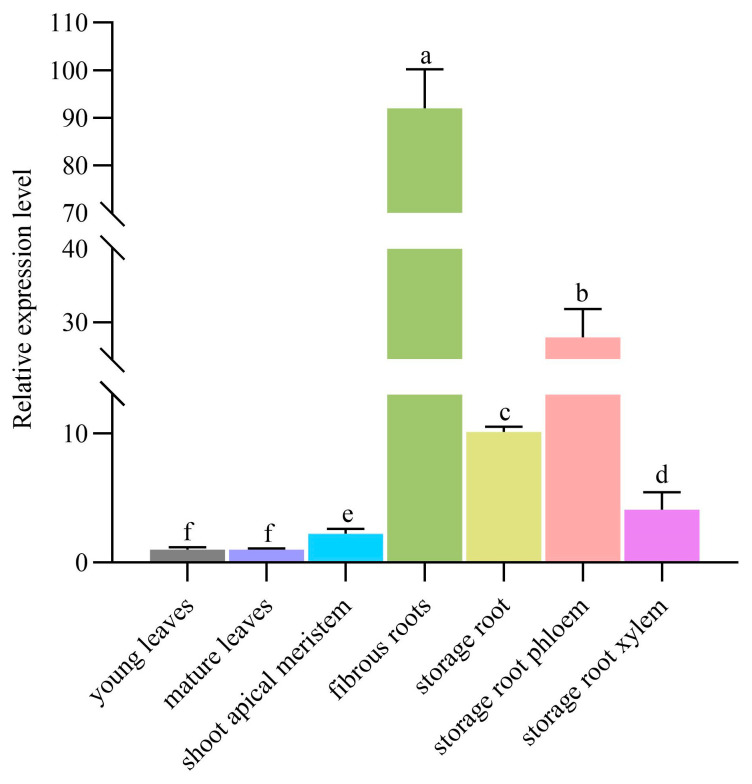
Expression analysis of the *MeSTP7* gene in cassava tissues. The storage roots were selected from cassava roots that had grown for 80 days. *MeTubulin* and *MeActin* were used as internal controls. The expression level of young leaves was set to 1. The data represent the mean ± standard deviation of three biological replicates. Different lowercase letters indicate significant differences (*p* < 0.05).

In order to study the expression pattern of *MeSTP7* in specific tissues of cassava, we used qRT-PCR to analyze *MeSTP7* gene expression in various cassava tissues, including young leaves, mature leaves, shoot apical meristem, fibrous roots, storage roots, storage root phloem, and storage root xylem. The results showed that *MeSTP7* expression was relatively low in young leaves, mature leaves, and shoot apical meristem but high in fibrous root and phloem of storage roots. Among these tissues, fibrous roots exhibited the highest expression level of *MeSTP7*, followed by the phloem of storage roots (Figure 1).

### 2.2. The Expression Pattern of MeSTP7Gene Under Hormone and Abiotic Stressor Treatments

**Figure 2:**
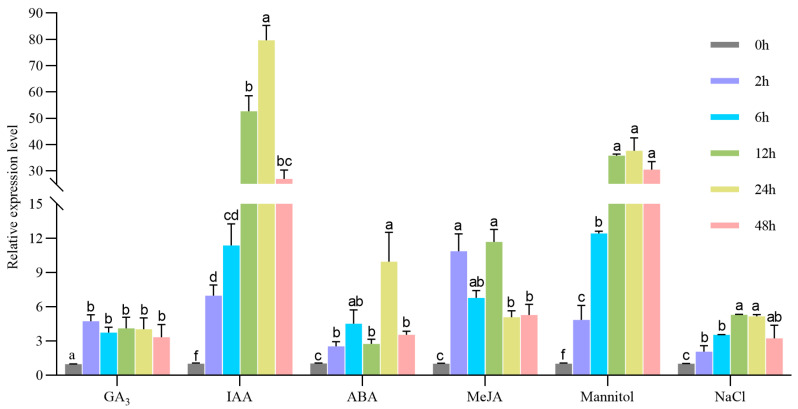
The root expression pattern of the *MeSTP7* gene under various treatments of cassava seedlings, including drought, salt, IAA, MeJA, ABA, and GA_3_. The treatment methods were as follows: gibberellin treatment was 100 μM GA_3_; IAA treatment was treated with 100 μM IAA; ABA treatment was 100 μM ABA; MeJA treatment was 100 μM MeJA; salt stress was 300 mM NaCl; the drought treatment was simulated by 100 mM mannitol. The *MeTubulin* gene and *MeActin* gene were normalized as an internal control. The relative expression value of the control sample was standardized to the control value 1. The data are expressed as the average of three independent organisms. Different lowercase letters indicate significant differences (*p* < 0.05).

In order to explore the role of *MeSTP7* in response to abiotic stressors and hormone signals, in this study, cassava seedlings were treated with exogenous hormones (GA_3_, ABA, IAA, and MeJA) and abiotic stressors (mannitol-simulated drought stress, NaCl salt stress). The expression pattern of *MeSTP7* was analyzed by qRT-PCR. The results showed that under treatment with GA_3_, IAA, ABA, MeJA, mannitol, and NaCl, the expression level of *MeSTP7* was upregulated to varying degrees (Figure 2). Among them, under IAA treatment, the expression of *MeSTP7* reached the most significant induction at 24 h (79.6-fold). Under GA_3_ treatment, the expression of *MeSTP7* reached the most significant induction at 2 h (4.7-fold). Under ABA treatment, the expression of *MeSTP7* reached the most significant induction (9.9-fold) at 24 h. Under MeJA treatment, the expression of *MeSTP7* reached the most significant induction (11.6-fold) at 12 h. Under drought stress, the expression of *MeSTP7* reached the most significant induction (37-fold) at 24 h. Under salt stress, the expression of *MeSTP7* reached the most significant induction at 12 h (5.3-fold). The results suggest that *MeSTP7* may respond to the stimulation by IAA, MeJA, ABA, GA_3_, salt, and simulated drought.

### 2.3. Transformation of MeSTP7 in Arabidopsis

**Figure 3:**
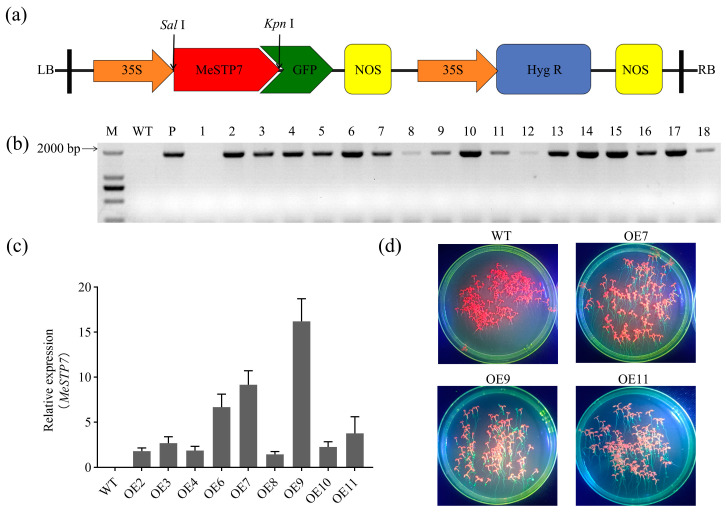
Detection of transgenic *Arabidopsis* expressing heterologous *MeSTP7*. (**a**) A schematic diagram of the pCAMBIA1300-*MeSTP7*: GFP recombinant vector. (**b**) PCR detection of the leaves of resistant transgenic *Arabidopsis* seedlings that survived on 1/2 MS medium supplemented with 100 mg/L hygromycin, T1 generation. M: DL2000; P: Positive plasmid control; 1–18: Transgenic *Arabidopsis* lines; WT: Columbia-0 *Arabidopsis*. (**c**) Expression of the *MeSTP7* gene in selected transgenic lines. The expression of OE8 was set to 1, and the AtActin gene was normalized as an internal control. (**d**) Expression of GFP in T3 transgenic lines was observed using a Luyor-3415RG dual-wavelength fluorescent protein excitation light source.

To reveal the function of *MeSTP7* in plant growth and development, we constructed a plant overexpression vector pCAMBIA1300-*MeSTP7*:GFP (Figure 3a). The vector was used to transform *Arabidopsis*. Following 100 mg/L hygromycin screening, 18 independent lines of positive transformants were obtained. These lines were designated as OE1-18, and 17 of them were confirmed by PCR analysis (Figure 3b). Nine lines with bright bands were selected for q-PCR analysis to detect the expression of the *MeSTP7* gene. Three lines with different expression levels were screened out: OE7, OE9, and OE11 (Figure 3c). Pure lines were further screened for subsequent experiments. Using a Luyor-3415RG dual-wavelength fluorescent protein excitation light source, green fluorescence was observed in OE7, OE9, and OE11 (Figure 3d), indicating that GFP was successfully expressed in transgenic plants. This also confirmed that *MeSTP7* overexpressing *Arabidopsis* had been obtained.

### 2.4. MeSTP7 Promotes the Early Growth of Arabidopsis

**Figure 4:**
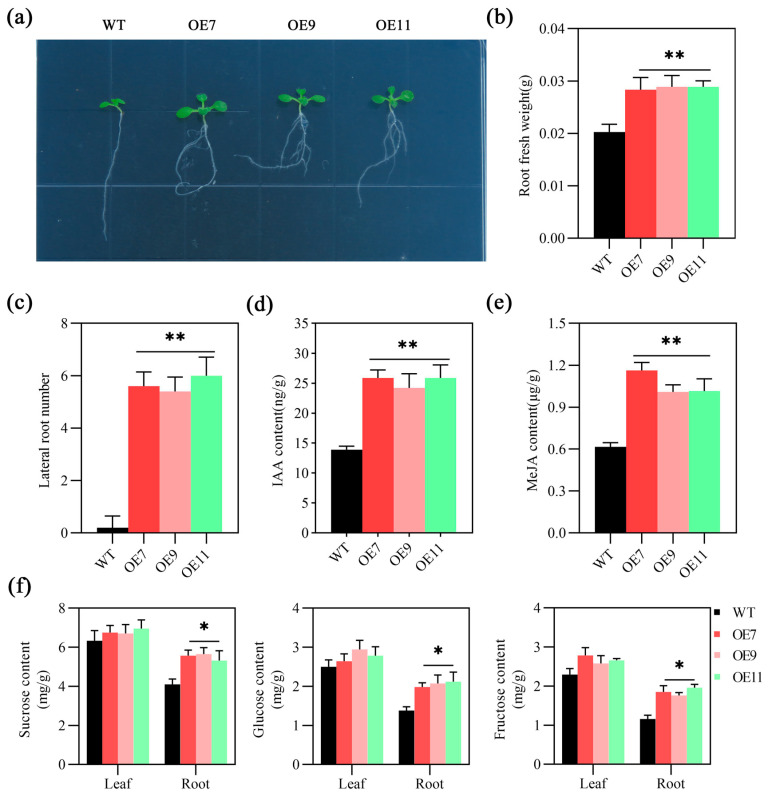
*MeSTP7* increases the levels of IAA, MeJA, sucrose, glucose, and fructose in *Arabidopsis* seedlings, promoting seedling growth. (**a**) Transgenic *MeSTP7* and WT plants were grown on 1/2 MS agar for 7 days. (**b**) Statistical analysis of plant root fresh weight. (**c**) Statistical analysis of the number of plant lateral roots. (**d**) The endogenous IAA content in the roots of the plants was measured. (**e**) The endogenous MeJA content in the roots of the plants was measured. (**f**) The content of glucose, fructose, and sucrose in WT and *MeSTP7*-overexpression *Arabidopsis* plants grown in 1/2 MS medium. The data were expressed as the mean ± SEM of three biological replicates. * represents a significant difference (*p* < 0.05), ** represents a highly significant difference (*p* < 0.01).

Phenotypic observations of *Arabidopsis* overexpressing *MeSTP7* at various growth stages revealed that the growth rate of these plants was significantly higher than that of the wild type (WT) at 7 days post-germination. Additionally, root fresh weight and lateral root number were markedly greater in *MeSTP7*-overexpressing *Arabidopsis* compared to WT (Figure 4a–c). We measured the levels of endogenous IAA and MeJA hormones, as well as sucrose, glucose, and fructose in *Arabidopsis*. Our results indicated that these levels were significantly higher in *MeSTP7*-overexpressing *Arabidopsis* compared to the WT (Figure 4d–f). At 15 days, *MeSTP7*-overexpressing *Arabidopsis* had more leaves than WT (Supplement Figure S1a). Before the 35-day bolting period, the leaf area and number of *Arabidopsis* were almost the same as WT. We weighed the root biomass at that time and found no significant difference (Supplementary Figure S1b–d). We observed no significant difference in flower and fruit morphology or fruit biomass at the flowering and podding stages (Supplement Figure S1f). These results indicate that the *MeSTP7* gene plays a significant enhancing role in the early growth characteristics of *Arabidopsis*. *MeSTP7*-overexpressing *Arabidopsis* had increased levels of IAA, MeJA, sucrose, glucose, and fructose and had greater root biomass and root morphological complexity compared to WT. However, the impact of *MeSTP7* on overall plant development becomes less pronounced in later growth stages.

### 2.5. The Number of Lateral Roots of Transgenic Arabidopsis Increased Under IAA Treatment

**Figure 5:**
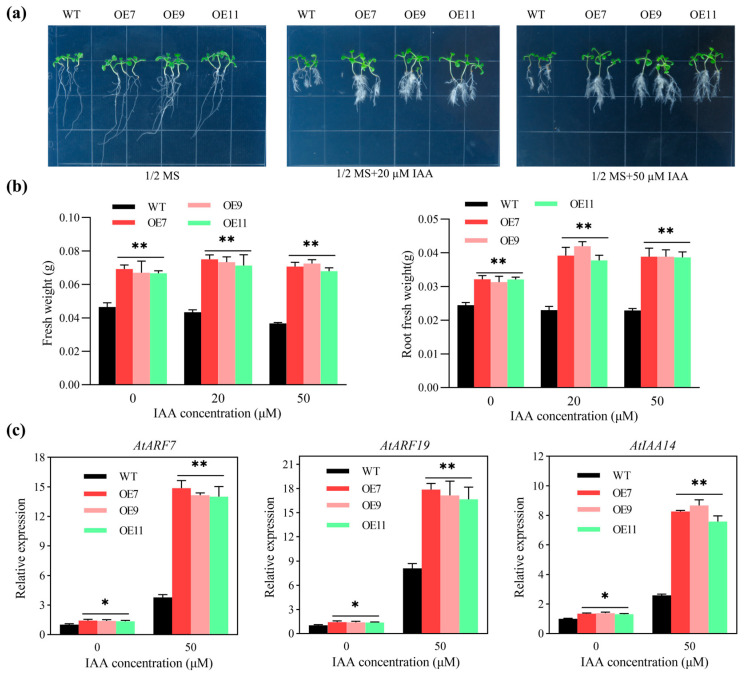
IAA treatment enhances root development in *Arabidopsis* with *MeSTP7* overexpression. (**a**) Phenotype of transgenic *Arabidopsis* under different IAA concentration gradients. (**b**) The total plant fresh weight and root fresh weight of *Arabidopsis* under different IAA concentration gradient treatments. (**c**) Expression of root development-related genes under different IAA concentrations. * represents a significant difference (*p* < 0.05), ** represents a highly significant difference (*p* < 0.01).

The fresh weight, root weight, and lateral root number of *MeSTP7* transgenic *Arabidopsis* (OE7, OE9, and OE11) were higher than those of WT on 1/2 MS medium without IAA. On the medium supplemented with 20 and 50 μM IAA, the transgenic plants exhibited significantly better root development, higher root fresh weight, and greater lateral root number than WT. As the IAA concentration in the medium increased, the roots of wild-type *Arabidopsis* initially increased but then decreased, with growth being inhibited, while the transgenic *Arabidopsis* was unaffected (Figure 5a,b).

Gene expression analysis showed that the auxin response factors *AtARF7*, *AtARF19,* and the auxin primary response gene *AtIAA14*, related to lateral root development, were significantly higher in transgenic lines than in WT on 1/2 MS medium without IAA. Under 50 μM IAA treatment, the expression levels of *AtARF7*, *AtARF19,* and *AtIAA14* increased in both transgenic and wild-type *Arabidopsis*, but the increase was greater in the transgenic lines than in WT (Figure 5c).

### 2.6. The Number of Lateral Roots of Transgenic Arabidopsis Decreased under MeJA Treatment

**Figure 6:**
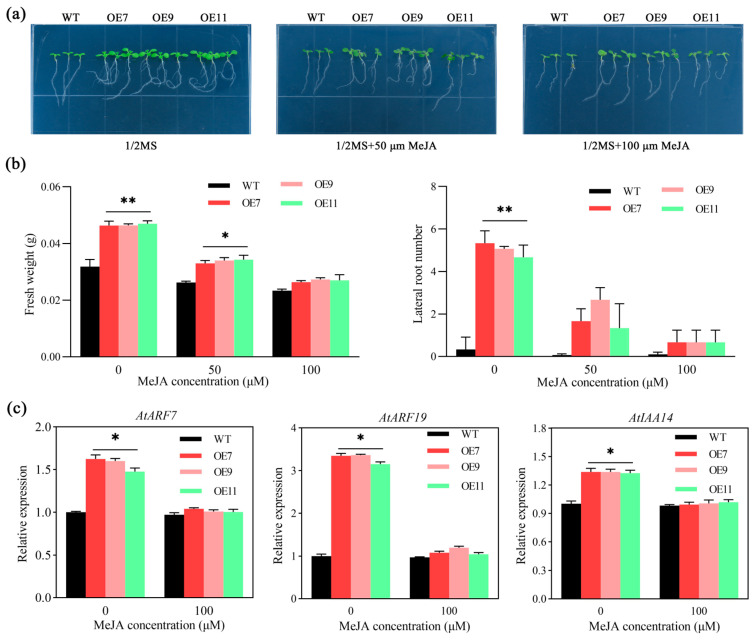
MeJA treatment inhibited the formation of lateral roots in *MeSTP7*-overexpressing *Arabidopsis* plants. (**a**) Phenotypes of transgenic *Arabidopsis* under different MeJA concentration gradients. (**b**) Fresh weight and lateral root number of transgenic *Arabidopsis* under different MeJA concentration gradients. (**c**) Expression of root development-related genes under different MeJA concentrations. * represents a significant difference (*p* < 0.05), ** represents a highly significant difference (*p* < 0.01).

Consistent with the results of 2.4, on 1/2 MS medium without MeJA, the total plant fresh weight, root weight, and lateral root number of *MeSTP7* transgenic *Arabidopsis* (OE7, OE9, and OE11) were higher than those of WT, and the roots were curved. On the medium supplemented with 50 and 100 μM MeJA, the strong root development ability of transgenic plant seedlings was weakened. Specifically, at 100 μM MeJA, the root weight and lateral root number were almost the same as those of WT, and the root curving effect was reduced (Figure 6a,b).

Gene expression analysis showed that the lateral root development-related genes *AtARF7*/19 and *AtIAA14* had significantly higher levels than WT on 1/2 MS medium without MeJA, which was consistent with the results of 2.5. Under 50 μM MeJA treatment, the expression of *AtARF7*/19 and *AtIAA14* in transgenic *Arabidopsis* was inhibited (Figure 6c). The results showed that the *MeSTP7* gene could induce the expression of *AtARF7*/19 and *AtIAA14*, while MeJA could inhibit them.

## 3. Discussion

In plants, sucrose synthesized in the leaves is transported to storage tissues for unloading via both the symplastic and apoplastic pathways to support cell division and growth in meristems [5,35]. In the apoplastic pathway, once sucrose enters the apoplast, it is hydrolyzed into glucose and fructose by cell wall invertase (CWINV). These monosaccharides are then transported into cells through monosaccharide transporters (STPs), providing energy for storing cells and synthesizing essential compounds (including protein, cellulose, and starch) [36]. Monosaccharides play a crucial role as carbon sources and sugar signals during plant growth and development [37]. Therefore, monosaccharide transport proteins play a key role in plant growth and development. The genes encoding STPs are primarily localized on the plasma membrane [5]. In cassava, most STPs function in actively dividing and extending early storage roots (after 30 and 40 days of growth) [38]. *AtSTP1* is highly expressed in germinating guard cells, seeds, and seedlings [12]; *AtSTP6*, *AtSTP8*, *AtSTP9*, *AtSTP10*, and *AtSTP11* are expressed in pollen tubes [39]; *AtSTP7* is expressed in the root tips of seedlings and in mature pollen and styles; *AtSTP8* is expressed in pollen, pollen tubes, ovules, and leaf veins; *AtSTP12* is expressed in pollen [17]; and *AtSTP13* is expressed in flowers, stems, young leaves, mature rosette leaves, roots, and throughout the seedling [11]. In cassava, the *MeSTPs* gene family is expressed in various tissues, including leaves, phloem, fibrous roots, and storage roots. *MeSTP7* shows high expression levels in the phloem and fibrous roots (Figure 1); *MeSTP15* has the highest expression level in fibrous roots; and *MeSTP17* and *MeSTP19* were highly expressed in early storage roots 80 days after planting [38]. In rice, 22 members of the *STPs* gene family have been identified, with the majority exhibiting peak expression during flowering, gradually decreasing as the rice heads and matures [40]. In alfalfa, most *MtSTPs* are predominantly expressed in root nodules, with only a few members expressed in stems and roots [41]. Our study indicates that the cassava *MeSTP7* gene has high expression levels during the early development of fibrous and storage roots (Figure 1). Under various hormonal treatments and abiotic stressor conditions, the transcription levels of *MeSTP7* exhibited significant changes (Figure 2), suggesting its important role in plant hormone response and abiotic stressor tolerance.

In studying the source-sink relationship, scientists have found that the transport sites and directions of sugars from source tissues to sink tissues are not fixed; they change according to different stages of growth and development [42]. Various organs of the plant have differing demands at different times, leading to changes in the functioning of transporters [43]. In the apoplastic pathway of sucrose unloading in the phloem, the activities of CWINVs and STPs dominate in storage tissues that are actively dividing and extending [44], whereas sucrose transport, primarily dominated by SUTs activity, prevails in storage tissues entering a storage mode [35,45,46]. Additionally, we found that the levels of sucrose, glucose, and fructose, as well as the endogenous hormones IAA and MeJA, were significantly elevated in the roots of transgenic *Arabidopsis* seedlings. This indicates that the expression of the *MeSTP7* gene accelerated monosaccharide transport in *Arabidopsis* seedlings, promoting sucrose metabolism and consequently increasing hormone levels. This also suggests that the *MeSTP7* gene functions during the rapid cell division and extension phases of transgenic *Arabidopsis* seedlings, while the role of the STPs correspondingly decreases as the plant enters a period of stabilized development.

Sugars are not only the primary energy source for plant life activities but also act as signaling molecules that interact with hormones to regulate the processes of plant growth and development [47,48]. The sensitivity to sugars at different developmental stages plays a crucial role in plant development, making sugar transport and signaling critical during these transitions [36]. Sugar signals work in concert with plant hormones to regulate growth; for instance, sucrose can modulate the metabolism, transport, and signaling of auxin, thereby controlling the elongation growth of meristematic tissues [49]. Transcription factors such as *bZIP1*, *bZIP11*, and *bZIP44* serve as negative regulators of auxin-mediated primary root growth and their expression is downregulated by sugars. Glucose interacts with the signaling and transport mechanisms of auxin to control the growth and development of seedling root systems [50]. In this study, we found that the *MeSTP7* gene was strongly induced by IAA, leading to an increase in lateral roots (Figure 5). Moreover, MeJA inhibited the formation of lateral roots in *MeSTP7*-overexpressing *Arabidopsis* plants (Figure 6), consistent with the role of MeJA as an auxin inhibitor that impedes lateral root formation [33,51]. ABA suppressed *MeSTP7*-induced lateral root formation by weakening the IAA response network in the root apical meristem [52]. Conversely, GA_3_ promoted the formation of lateral roots induced by *MeSTP7* (Supplementary Figure S3). Previous studies have indicated that GA_3_ may enhance root development in *Arabidopsis* by regulating IAA transport and signaling [53]. Our findings demonstrate that *MeSTP7* promotes the development of lateral roots in *Arabidopsis* seedlings, with its expression being induced by multiple hormones. Notably, IAA strongly induced *MeSTP7* expression, while MeJA treatment inhibited it. Hormonal treatments of transgenic *Arabidopsis* revealed that IAA and GA_3_ stimulated the development of lateral roots, while MeJA and ABA suppressed it. This indicates that IAA, GA_3_, MeJA, and ABA collectively induce and regulate the formation of lateral roots in *Arabidopsis* seedlings.

It is widely accepted that the development of lateral roots in *Arabidopsis* relies on regulation by LR/*IAA14*–*ARF7* (*ARF19*) [54,55]. Under optimal growth conditions, plants utilize most of the carbohydrates produced through photosynthesis to support their growth. To achieve a balanced growth between aerial parts and root systems, auxin synthesized in the aerial parts is transported to the root apical meristem via auxin efflux carriers (such as PIN and NRT), thereby inducing the growth of the root apical meristem and enhancing the growth rate of the roots. Our quantitative analysis revealed that the transcription levels of *IAA14* were significantly elevated in transgenic *Arabidopsis*, along with high levels of auxin response factors *ARF7* and *ARF19*. Additionally, the transcription factor *LBD16/29*, regulated by *ARF7*, was also found to be highly expressed, as were the IAA homeostasis regulators *WOX5/7* (Supplementary Figure S4). Furthermore, the expression levels of nitrate transporters and glutamine synthetase, which provide amino acids for root development, were significantly upregulated. Based on these results, we hypothesize that the overexpression of *MeSTP7* increases the concentration of hexoses in the root system of *Arabidopsis* seedlings. This elevated sugar concentration activates *ARF7*/19, leading to an upregulation of IAA synthesis, promoting the transport of auxin to the root apical meristem and consequently activating the formation of lateral roots.

## 4. Materials and Methods

### 4.1. Analysis of the Tissue-Specific Expression Pattern of MeSTP7 in Cassava

*Manihot esculenta* Crantz SC8 is cultivated in Lingao County, Hainan Province, China. Under normal conditions, young leaves, mature leaves, shoot apical meristems, adventitious roots, and storage roots were collected from field plants 80 days after planting. Three leaves were taken from both the young and mature leaves. For the shoot apical meristem, adventitious roots, and storage roots, 10 g was collected from each tissue. The storage roots were dissected with a sterile scalpel to separate the phloem and xylem, with 10 g taken from each tissue and then quickly frozen in liquid nitrogen. RNA was extracted from the aforementioned tissues using the RNA Plant Plus reagent (Tiangen, Beijing, China) according to the manufacturer’s instructions. cDNA was synthesized using the PrimeScript™ RT kit (TaKaRa, Dalian, China), and the tissue-specific expression level of *MeSTP7* was analyzed with the TaKaRa SYBR^®^ Premix Ex Taq™ II reagent (TaKaRa, Dalian, China). *MeTubulin* and *MeActin* were used as internal controls.

### 4.2. Hormonal and Abiotic Stressors Treatments for Cassava

Seedlings of the SC8 variety were cultivated in a climate-controlled growth chamber located in Haikou, China, under conditions set at a temperature of 28 °C with a 16 h daily light cycle and a humidity level of 60%. Thirty-day-old seedlings were excised from the Murashige and Skoog (Coolaber, Beijing, China) medium and subjected to treatments with 100 mM mannitol and 300 mM NaCl solutions at intervals of 0, 2, 6, 12, 24, and 48 h. The 30-day-old seedlings were also treated with 100 µM GA_3_, 100 µM IAA, 100 µM ABA, and 100 µM MeJA solutions at the same time intervals. After each treatment, samples of the roots, stems, and leaves were collected, rapidly frozen in liquid nitrogen, and subsequently stored at −80 °C for future RNA extraction.

### 4.3. Construction of MeSTP7 Plant Overexpression Vector

The pCAMBIA1300 vector was used as the backbone vector, which contains dual 35S promoters and the hygromycin resistance gene Hyg R. The primers p1300-*MeSTP7*-F and p1300-*MeSTP7*-R were designed by using SnapGene4.2.4 software for PCR amplification, and the coding region fragment of the *MeSTP7* gene containing the cleavage site was obtained. Using the double-enzyme digestion method, the target gene was inserted into the plant overexpression vector pCAMBIA1300 to construct the pCAMBIA1300-*MeSTP7*:GFP vector. The recombinant vector was transformed into *E. coli* (DH5α), and the monoclonal plasmid was picked up for preliminary enzyme digestion verification. The correct plasmid was sent to Sangon Biotech (Shanghai) for sequencing, and the detection primers used were 1300-F and 1300-R. The recombinant plasmid pCAMBIA1300-*MeSTP7*:GFP was introduced into *Agrobacterium tumefaciens* LBA4404 competent cells. The primers are listed in Table S1.

### 4.4. Genetic Transformation and Identification of MeSTP7-Overexpression Arabidopsis Transgenic Lines

The transgenic *Arabidopsis* lines were generated by an *Agrobacterium*-mediated transformation method. The *Agrobacterium* solution containing pCAMBIA1300-*MeSTP7*:GFP plasmid was cultured, and the inflorescences of *Arabidopsis* were infected by the floral dip method. The T0 seeds were collected and vernalized at 4 °C for 2 days after drying. The positive transformants were screened by culturing on 1/2 MS (containing 100 mg/L hygromycin) solid medium. The *Arabidopsis* seedlings of various lines capable of rooting on hygromycin-containing medium were identified by leaf PCR with 2× M5 HiPer Superluminal mix with blue dye (Mei5bio, Beijing, China) and the detection primers 1300-F and 1300-R. A Luyor-3415RG (LUYOR, Joliet, IL, USA) dual-wavelength fluorescent protein excitation light source was used to observe whether the transgenic plants (T1) emitted green fluorescence, and the fluorescence intensity was used to qualitatively detect *MeSTP7* expression. Three transgenic lines with strong green fluorescence were selected for subsequent experiments. In order to evaluate the expression level of *MeSTP7* in transgenic *Arabidopsis*, total RNA was isolated from transgenic *Arabidopsis* lines (T3) OE7, OE9, OE11, and wild-type *Arabidopsis*, and RNA was reversed to cDNA. SYBR^®^ Premix Ex Taq^TM^ II reagent (TaKaRa, Dalian, China) was used to analyze the expression level of each gene. The *Arabidopsis* actin gene was employed as an internal control. The 2^−∆∆^Ct method was used to calculate the relative expression level. Each sample of the experiment had three biological replicates, and the calculated results were plotted with Excel. The primer pairs used were as follows: q*MeSTP7*-F/R and qAtActin-F/R. The primers q*MeSTP7*-F/R and qAtActin-F/R are listed in Table S1.

### 4.5. Phenotypic Observation of Transgenic Arabidopsis at Different Growth Stages

The seeds of three T3 transgenic *Arabidopsis* lines, OE7, OE9, OE11, and wild-type *Arabidopsis*, were vernalized and sown in whole vermiculite. After 10 days of growth, *Arabidopsis* plants with the same growth vigor were selected and transplanted into a small pot (7 cm × 7 cm). One plant was planted in each pot, and the growth was recorded every 7 days until the harvest. Five biological replicates were measured for each line.

### 4.6. Hormonal Treatment of Transgenic Arabidopsis

Under sterile conditions, three T3 transgenic *Arabidopsis* lines, OE7, OE9, OE11, and wild-type *Arabidopsis*, were disinfected, vernalized, and grown on 1/2 MS medium for 6 days. Then, the plants with two true leaves were transplanted to a 10 cm × 10 cm square plate, treated with different concentrations of hormones, and cultured in an environment with temperatures of 22 ± 1 °C, 60% humidity, and a 16 h light/8 h dark cycle. The hormone treatment involved sterilizing the 1/2 MS medium, adding hormones after the medium cooled to below 50 °C, and then transplanting *Arabidopsis* into the 1/2 MS medium containing hormones for growth. The phenotype was observed after 7 days. The IAA treatment medium was 1/2 MS supplemented with 0, 20, and 50 μM IAA. The MeJA treatment medium was 1/2 MS supplemented with 0, 50, and 100 μM MeJA. The ABA treatment medium was 1/2 MS supplemented with 0, 50, and 100 μM ABA. The medium for GA_3_ treatment was 1/2 MS supplemented with 0, 50, and 100 μM GA_3_. In this experiment, three biological replicates were carried out.

### 4.7. Determination of Endogenous Hormone Content in Transgenic Arabidopsis

Extraction method: The analysis for IAA and MeJA was conducted by Suzhou Keming Biotechnology Co., Ltd. (Suzhou, China). IAA in plant samples was extracted using pre-cooled 80% methanol, followed by decolorization with chloroform, pH adjustment, and ethyl acetate extraction, then concentrated and dissolved in methanol for analysis. MeJA was extracted with 80% acetonitrile, purified, and eluted through a C18 column, then concentrated and dissolved in methanol for analysis.

HPLC conditions: The Rigol L3000 high-performance liquid chromatography system equipped with a Sepax-C18 column was used. For IAA, the mobile phase consisted of water, acetic acid, and methanol (ratio 60:6:40), with detection wavelengths set at 275 nm (excitation) and 345 nm (emission). For MeJA, the mobile phase consisted of 0.1% phosphoric acid in water (A) and acetonitrile (B) in a 4:6 ratio, with a detection wavelength of 210 nm.

### 4.8. RNA Extraction and qRT-PCR Analysis of Related Genes

Total RNA was isolated from the treated transgenic *Arabidopsis* lines OE7, OE9, OE11 and wild-type *Arabidopsis* using the RNA Plant Plus reagent (TianGen, Beijing, China) based on the manufacturer’s instructions. TaKaRa SYBR^®^ Premix Ex TaqTM II reagent (TaKaRa, Dalian, China) was used to analyze the expression level of root development-related genes in *Arabidopsis*. The *Arabidopsis* actin gene was employed as an internal control. The 2^−∆∆^Ct method was used to calculate the relative expression level. Each sample of the experiment had three biological replicates, and the calculated results were plotted with Excel. The primer pairs used are shown in Table S1.

### 4.9. Statistical Analysis

The data are presented here as the mean ± SD, and the data from three independent experiments were analyzed with a one-way analysis of variance. The value of *p* ≤ 0.05 was considered significant using GraphPad Prism 8 software.

## 5. Conclusions

We studied the tissue expression pattern of *MeSTP7* in cassava and the expression pattern of *MeSTP7* under hormone treatment and abiotic stressors. It was found that *MeSTP7* was highly expressed in the phloem and fibrous roots of the early storage roots of cassava, and the expression of *MeSTP7* was most strongly induced by IAA. *MeSTP7* was heterologously expressed in *Arabidopsis*, and it was found that *MeSTP7* promoted the growth of *Arabidopsis* seedlings at an early stage. The contents of IAA, MeJA, sucrose, glucose, and fructose in the roots of transgenic *Arabidopsis* seedlings increased significantly. Under IAA treatment, the number of lateral roots of transgenic seedlings increased significantly. Under MeJA treatment, the number of lateral roots of transgenic *Arabidopsis* seedlings decreased significantly. The results of qRT-PCR showed that the expression of *IAA14*, *ARF7,* and *ARF19*, which are important regulators of lateral root development, was significantly increased in transgenic plants and induced by IAA, while MeJA inhibited their expression. Based on the above results, we conclude that *MeSTP7* acts in the roots of *Arabidopsis*, accelerating the unloading of sucrose and rapidly increasing the concentrations of sucrose, glucose, and fructose in the tissues. This, in turn, alters the endogenous hormone levels of IAA and MeJA in the roots of *Arabidopsis*, thereby promoting the development of *Arabidopsis* seedlings.

## Data Availability

All analyzed data for this study are included in the contents of this article and Supplementary Materials.

## Supplementary Materials

The following supporting information can be downloaded at: https://www.mdpi.com/article/10.3390/plants13213102/s1, Table S1: Primers used in experiments; Figure S1: Phenotypes of *Arabidopsis* with Overexpression of *MeSTP7* at Different Growth Stages; Figure S2: The response of *MeSTP7*-overexpressing *Arabidopsis* to ABA; Figure S3: The response of *MeSTP7*-overexpressing *Arabidopsis* to GA_3_; Figure S4: qRT-PCR analysis of the expression of *AtLBD16*, *AtLBD29*, *AtWOX5,* and *AtWOX7* in transgenic *Arabidopsis* lines.
