# Supplementary material for: Overexpression of Cassava MeSTP7 Promotes Arabidopsis Seedling Development
Source: Plants (Basel). 2024 Nov 4;13(21):3102. doi: 10.3390/plants13213102 (PMC11548149; doi:10.3390/plants13213102)
Supplement: Supplementary file 1 [file plants-13-03102-s001.zip › plants-3224733-supplementary.pdf]

Supplementary Material

Table S1. Primers used in experiments.

Name	Upstream primer (5'-3')	Downstream primer (5'-3')
pCAMBIA1300-MeSTP7	AGCGTCGACATGACGGGTTTTGCCATTA	CGCGGATCCACCATGAAGAGAGGCATA
pCAMBIA1300-DNA	GTTGATACATATGCCCGTCG	CTCGCCCTTGCTCACCAT
MeSTP7-qPCR	GGCGGTGCCTCTATTTCTATC	TATGCCGATGGTGACGTTAAG
MeTubulin-qPCR	GTGGAGGAACTGGTTCTGGA	GTGGAGGAACTGGTTCTGGA
MeActin-qPCR	CGTTTGTGGGAATGGAAGCT	TTGCTCATACGGTCAGCGATA
AtIAA14-qPCR	AAGCCTCCTGCTAAAGCACA	CCATCCATGGAAACCTTCAC
AtARF7-qPCR	GTCACAGTGAGCAAGTCGCG	GACCCATGTCAGAAGCAAGC
AtARF19-qPCR	CAAGTTCCAACGAAGGAGAGA	ACCACAAGACTTCCCACAGG
AtActin-qPCR	TTCCTCATGCCATCCTCCGTCT	TTGCTCATACGGTCAGCGATA
AtLBD16-qPCR	TGACCCTGTTTATGGATGTGTC	TGATTGCAAGAAAGCCACCT
AtLBD29-qPCR	GCTAGGCTTCAAGATCCCATC	TGTGCTGCTTGTTGCTTTAGA
AtWOX5-qPCR	CTCCAACTCCAAGGTGGACA	ATGGCGGTGGATGTTCCATT
AtWOX7-qPCR	AACAACAACGGAGGAGGAGG	CAGTCCCGCCTTGAACAGAT

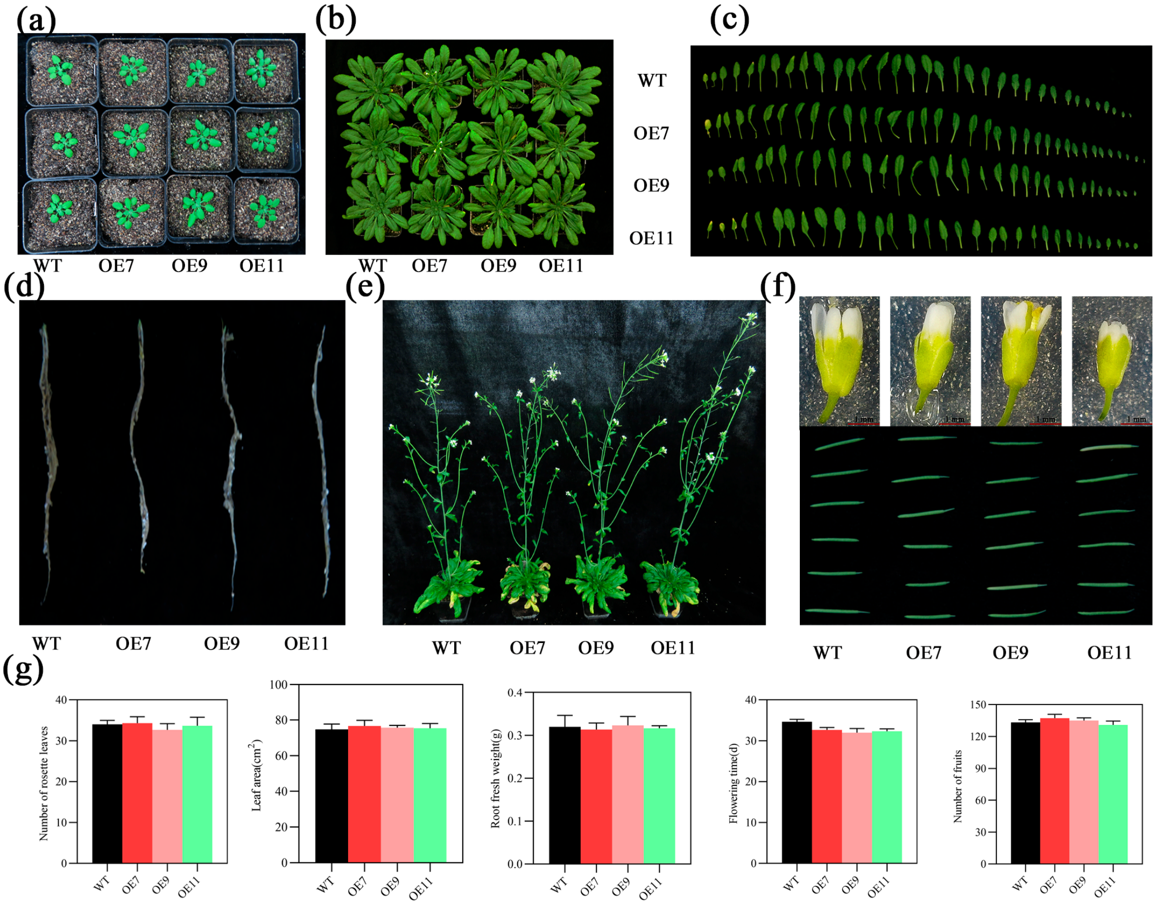

Figure S1. Phenotypes of the *MeSTP7* overexpressing *Arabidopsis* at various growth periods. a: *MeSTP7* overexpression *Arabidopsis* seedlings phenotype; b, c: *MeSTP7* overexpression *Arabidopsis.* bolting phenotype; d: Root of *MeSTP7* overexpression *Arabidopsis*; e, f: *MeSTP7* overexpressed *Arabidopsis* mature phenotype; g: Phenotypic statistics of *MeSTP7* overexpression *Arabidopsis.*

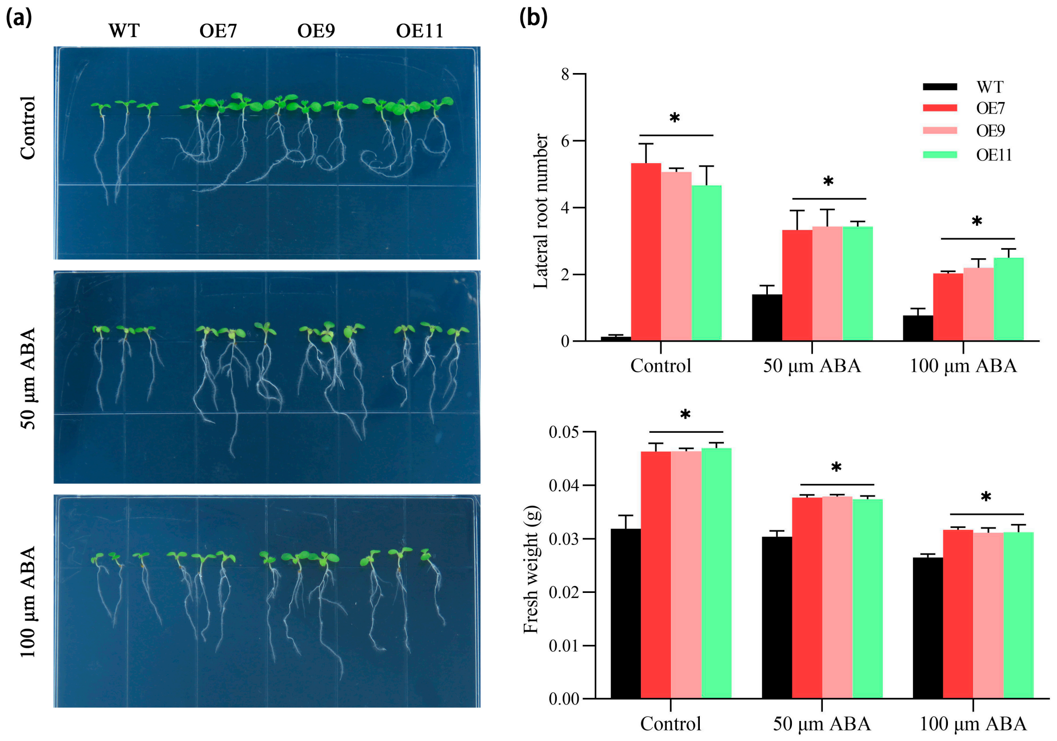

Figure S2. The response of MeSTP7-overexpressing *Arabidopsis* to ABA. (a) Phenotype of transgenic *Arabidopsis* under different ABA concentration gradients. (b) The fresh weight and lateral root number of transgenic *Arabidopsis* under different ABA concentration gradients.

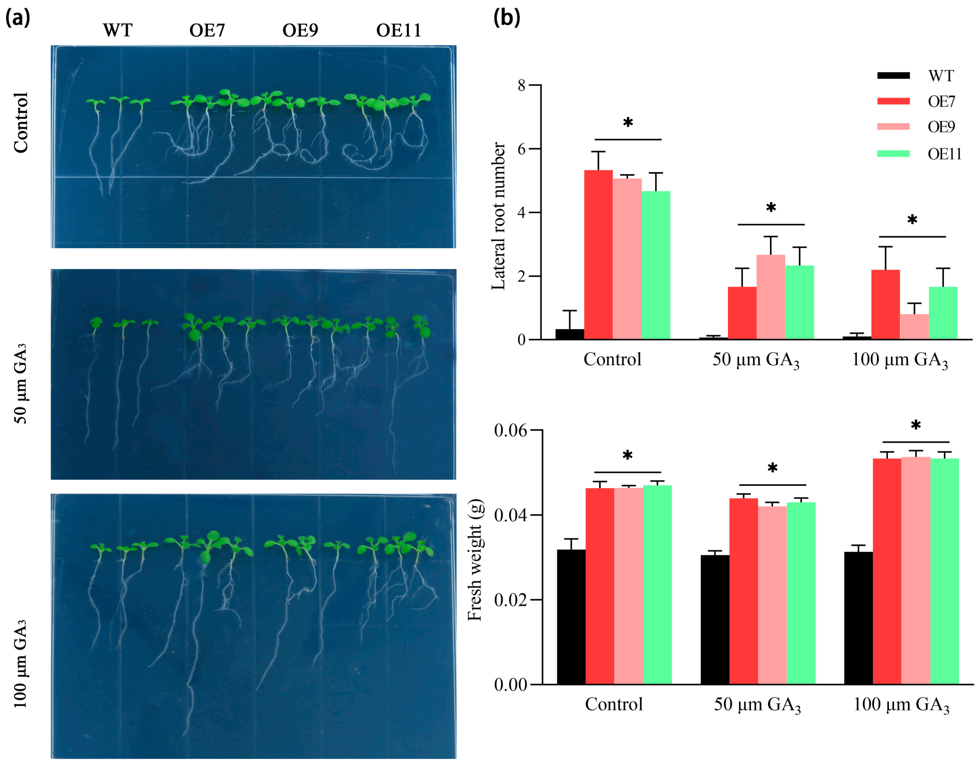

Figure S3. The response of MeSTP7-overexpressing Arabidopsis to GA_3. (a) Phenotype of transgenic Arabidopsis under different GA_3 concentration gradients. (b) The fresh weight and lateral root number of transgenic Arabidopsis under different GA_3 concentration gradients.

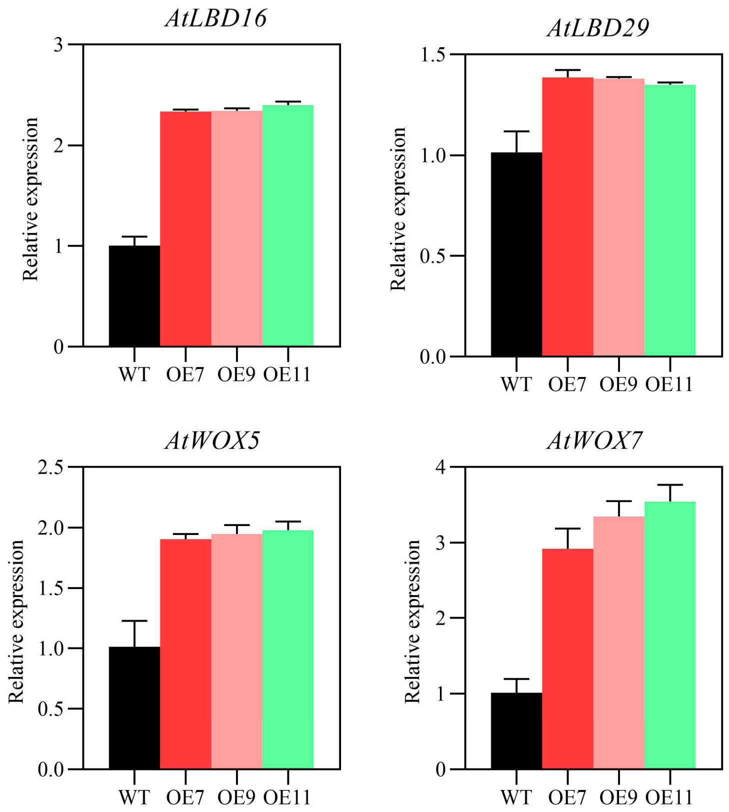

Figure S4. qRT-PCR analysis of the expression of AtLBD16, AtLBD29, AtWOX5 and AtWOX7 in transgenic *Arabidopsis* lines.
